# Hematological Parameters and Growth Curves for Captive Olive Baboons (*Papio anubis*): Effects of Viral Status, Sex, Age, and Rearing Status

**DOI:** 10.1111/jmp.70050

**Published:** 2025-12-04

**Authors:** Sarah J. Neal, Shannon Whitney, Elizabeth Magden, Joe H. Simmons

**Affiliations:** ^1^ The University of Texas MD Anderson Cancer Center Michale E. Keeling Center for Comparative Medicine and Research Bastrop Texas USA; ^2^ Department of Biology Texas State University San Marcos Texas USA

**Keywords:** baboon, chemistry, growth, hematology, interval, reference

## Abstract

**Background:**

Baboons are valuable models for human health research, yet existing hematology, serum chemistry, and growth reference data are limited by small sample sizes, narrow age ranges, and inconsistent reporting.

**Methods:**

This study addresses these gaps by analyzing data from 848 captive olive baboons to establish comprehensive reference intervals and growth curves. We examined variations in hematology and serum chemistry across age, sex, rearing history (mother‐ vs. nursery‐reared), and viral status (specific pathogen free vs. conventional).

**Results:**

Significant differences were found in 58.8% (10/17) of hematology and 37% (10/27) of chemistry parameters between SPF and conventional baboons, as well as across age, sex, and rearing categories. Growth curves aligned with previous studies but showed higher weight ranges.

**Conclusions:**

While many differences were statistically significant, not all may be clinically relevant. These findings provide a robust resource for veterinary care and biomedical research involving baboons.

## 1 Introduction

Baboons serve as model systems in biomedical research for a variety of human health‐related diseases and disorders [1, 2, 3, 4, 5, 6, 7, 8]. Reference intervals (RIs) for blood chemistry and hematology are needed for both veterinary care as well as interpretations of the effects of various treatments within such models. Furthermore, the specific model may necessitate the need for reference intervals of specific age, sex, or viral status categories of animals. For example, baboon research models for human reproduction will require reference intervals for adult females, whereas a xenotransplantation study will likely utilize reference intervals from baboons that are free of specific pathogens that can cause complications under immunosuppressive conditions, and a study examining the effects of aging on brain function will require reference intervals from adult and geriatric baboons.

The definitive source for reference ranges for baboons in laboratory animal medicine includes several sets of reference ranges for baboons [9]. These include hematological reference ranges (mean and 2 standard deviation range) separated by sex and housing type, as well as serum chemistry reference ranges separated by sex. These ranges are reported for *Papio* spp., which combines several species subtypes (e.g., hamadryas: *Papio hamadryas* ; olive: *Papio anubis* ; and yellow: *Papio cynocephalous*). The sample sizes across hematological parameters in these groups range from 30 to 90 individuals, but the chemistry reference ranges include sample sizes of just 25 (15 males and 10 females). Such small sample sizes increase the risk for individual datapoints to present undue influence within the dataset and resulting range, particularly when relying on mean and standard deviation as the measures of central tendency and variation [10, 11]. Importantly, these reference ranges do not include categorizations by viral status, rearing history, or age group.

There are several additional published sources for baboon reference ranges, intervals, and standards. One includes hematology and blood chemistry reference standards for 110 infant baboons of two species (olive and hamadryas combined, as the authors did not find significant differences as a function of species) [12]. Another includes only a select number of blood chemistry parameters for 29 free‐ranging olive baboons [13]. Yet another reports hematological and chemistry values (mean ± SD) for 33 male and female baboons used in xenotransplantation studies [14]. Although one study showed no significant differences in blood chemistry and hematology parameters as a function of SPF status, this study was limited due to a small sample size of juvenile SPF baboons [15]. Furthermore, viral infection in non‐SPF primates may produce certain immunologic deficits, which can impact RI development and interpretation [16]. While these articles provide useful information, the results may be affected by small sample sizes and the inclusion of subsets of populations and/or parameters. Given that research and medicine rely on the interpretation of results in the context of what is normal for a healthy animal, a more comprehensive and robust dataset based on moderate to large sample sizes is needed to enhance the baboon research model and quality of care.

Another metric or reference measure used in research and veterinary care that would benefit from more comprehensive data is growth curves, a reference curve showing typical patterns of weight change across age, providing age‐specific reference points. A number of baboon growth curves across settings exist, but they suffer from relatively small sample sizes and/or limited age ranges. For example, Leigh [17] reports body mass growth curves for a large sample (*n* = 457), but the data are collapsed across baboon subspecies and hybrids, and only include baboons up to 15 years of age, thereby excluding the category of geriatric baboons. Another study includes age and sex specific growth rates in chacma baboons, but with subset sample sizes as low as 5 individuals, and an age range of just 0–8.5 years of age [18]. Strum [19] reported on the relationship between weight and age in wild olive male and female baboons up to 24 years of age, but this was published 35 years ago, and does not capture how growth may differ between wild and captive populations. As such, similar to reference interval data, larger sample sizes and the inclusion of expanded age ranges are needed to better define the baboon resource.

The aim of this article is to provide comprehensive reference intervals and growth curves for the research and veterinary communities with baboons under their care. We did not aim to test hypotheses, but rather aimed to explore and characterize hematological and serum chemistry parameters. To accomplish this, we used recent, archival data from a large sample of baboons housed at the SPF18 Baboon Research Resource (SPF18BRR) in Bastrop, Texas, to develop hematological and chemistry reference intervals using accepted and widely used statistical guidelines [11]. We also aim to answer whether reference ranges differ based on age, sex, rearing, and viral status categories.

## 2 Materials and Methods

### 2.1 Humane Care Guidelines

The authors confirm that the ethical policies of the journal, as noted on the journal's author guidelines page, have been adhered to and the appropriate ethical review committee approval has been received. The US National Research Council's guidelines for the Care and Use of Laboratory Animals were followed. All research and experimental protocols complied with those approved by the UTMDACC Institutional Animal Care and Use Committee (ACUF # 1665‐RN01) and complied with the legal requirements of the United States and the ethical guidelines put forth by AALAS, the Animal Welfare Act, and The Guide for the Care and Use of Laboratory Animals. The Keeling Center has been fully accredited continuously since 1979 by AAALAC.

### 2.2 Subjects

We used indirect sampling for the development of reference intervals and growth curves (i.e., utilizing results from our medical database and using statistical methods to eliminate outliers [11]). Subjects for body weight analyses included a total of 848 captive olive baboons (446 females, 402 males, Table 1) housed at the Michale E. Keeling Center for Comparative Medicine and Research at The University of Texas MD Anderson Cancer Center in Bastrop, Texas. Baboons were housed in outdoor corrals or Primadomes with inside access, or in indoor/outdoor runs, in two separate colonies on campus. The first colony is the Specific Pathogen Free‐18 (SPF‐18) colony (a colony of baboons that are free of 18 specific viruses and pathogens), and the second is the conventional colony (non‐virus‐free). Baboons were housed in breeding groups consisting of one or two breeding males, 12–16 breeding females, and, in the SPF colony, their juvenile and infant offspring (0–3 years of age). Across both colonies, baboons ranged in age from 0 to 23.25 years (mean age = 4.25 years, standard deviation = 4.94 years). The blood chemistry and hematology data were available for a subset of 530 baboons (57% female). Across the sample used for RIs, baboons ranged in age from 0 to 22.3 years old, including 96 infants (0–12 months of age), 302 juveniles (aged 1–4 years), 99 adults (5–14 years), and 33 geriatric individuals (15+ years).

**TABLE 1:** Sample sizes across SPF status, sex, rearing (MR, mother‐reared; NR, nursery‐reared; Unk, unknown), and age group (years).

		Hematology and chemistry analyses				Growth curves					
		Female		Male		Female			Male		
		MR	NR	MR	NR	MR	NR	Unk	MR	NR	Unk
SPF	Infant (0–12 mos)	27	15	35	19	50	19	17	66	28	21
	Juvenile (1–4 years)	73	73	65	88	86	48	11	109	100	38
	Adult (5–14 years)	44	30	9	9	68	57	5	11	12	0
	Geriatric (15+ years)	1	8	1	1	7	21	1	2	3	0
Conventional	Infant (0–12 mos)	0	0	0	0	0	1	0	0	2	0
	Juvenile (1–4 years)	0	3	0	0	0	1	2	0	0	3
	Adult (5–14 years)	3	4	0	0	4	5	9	3	1	0
	Geriatric (15+ years)	20	1	0	1	29	4	1	1	1	1

Nursery‐reared individuals were defined as those baboons that were separated from the dam within 24 h following birth and cared for by humans, raised in an incubator with access to infant formula until they were put into small, same‐age peer social groups starting at 3 weeks with increasing social exposure until 2 years of age, when they were introduced to larger adult and sub‐adult social groups. Mother‐reared individuals were defined as baboons that were not separated from their dam for at least the first 6 months of life and were reared in their natal group during that time.

Blood values were taken from hematology and chemistry records obtained during routine biannual physical exams conducted between 2017 and 2025. One timepoint per baboon was used for analyses. Additionally, animals that were injured, recovering from an injury, or were pregnant were excluded from analyses. Body weights in kilograms (kg) were also obtained during these biannual physical exams while baboons were sedated. The sedation process has been described previously [20]. Briefly, baboons were fasted for 12 h, sedated using a ketamine injection, and blood was collected when the animal was sedated. Blood was collected following immobilization by intramuscular injection of ketamine HCl (10 mg/kg) and anesthetization with isoflurane inhalation (1%–3%) per standard institutional guidelines. Blood samples were collected from the femoral vein in K2EDTA‐treated and serum separator vacuum tubes (Becton Dickinson, Franklin Lakes, NJ) for hematology and serum chemistry analysis, respectively. Hematology and serum chemistry analyses were performed on the same day as the collections. Although some research has shown that ketamine administration can affect hematology values (e.g., lowered CBC counts), the variation within individuals and groups has been found to be higher than the variation between anesthetized and unanesthetized groups, indicating that the biological significance of the effects of ketamine on hematology may be slight [21].

Hematology analysis was performed using a Siemens Advia 120 multi‐parameter automated hematology analyzer (Siemens, Tarrytown, NY). The analyzer was calibrated and validated for baboons. Parameters included: red blood cell count (RBC, 10^6^/μL), hemoglobin (HGB, g/dL), hematocrit (HCT, %), mean corpuscular volume (MCV, fl), mean corpuscular hemoglobin (MCH, pg), mean corpuscular hemoglobin concentration (MCHC, g/dL), red blood cell distribution width (RDW, %), white blood cell count (WBC, 10^3^/μL), absolute neutrophil count (NEUT, 10^3^/μL), absolute lymphocyte count (LYMP, 10^3^/μL), absolute monocyte count (MONO, 10^3^/μL), absolute eosinophil count (EOS, 10^3^/μL), absolute basophil count (BASO, 10^3^/μL), relative neutrophil percentage (NEUT %), relative lymphocyte percentage (LYMP %), relative percentage monocyte (MONO %), relative eosinophil percentage (EOS %), relative basophil percentage (BASO %), mean platelet count (PLT, 10^3^/μL), and platelet volume (MPV, fl).

Serum chemistry analysis was performed using a Beckman‐Coulter AU680 chemistry analyzer (Beckman Coulter, Brea, CA). The analyzer was calibrated and validated for baboons. Blood was allowed to clot at room temperature for 30–60 min before centrifugation at 1300 *g* for 10 min to obtain serum. Measured parameters included alanine aminotransferase (ALT, IU/L), alkaline phosphatase (ALP, IU/L), aspartate aminotransferase (AST, IU/L), gamma‐glutamyltransferase (GGT, IU/L), total bilirubin (TBIL, mg/dL), lactate dehydrogenase (LDH, IU/L), creatine kinase (CK, IU/L), blood urea nitrogen (BUN, mg/dL), creatinine (CREA, mg/dL), calcium (CA, mg/dL), phosphorus (PHOS, mg/dL), glucose (GLUC, mg/dL), sodium (NA, mEq/L), potassium (K, mEq/L), chloride (CL, mEq/L), total carbon dioxide (TCO2, mEq/L), anion gap (AGAP, mEq/L), osmolality (OSMO, mOsm/kg), total protein (TP, g/dL), albumin (ALB, g/dL), globulins (GLOB, g/dL), albumin/globulin ratio (AGR, g/dL), cholesterol (CHOL, mg/dL), triglycerides (TRIG, mg/dL), iron (FE, μg/dL), total iron binding capacity (TIBC, μg/dL), and unbound iron blood count(UIBC, μg/dL).

### 2.3 Data Analysis

We used methods described by [11] as a guide for our statistical approach. We first examined histograms and Q–Q plots to assess for normality, which showed that several variables showed non‐Gaussian distributions. We then removed outliers from the dataset using Tukey's method, in which observations that fall between the inner and outer fences in each direction are “far out” outliers, while those that fall below the outer fence F1 or above the outer fence F3 are “extreme” outliers [22]. We report which outliers were removed in Table 2. Following outlier removal, we calculated the mean, standard deviation (SD), median, and 95% reference interval for each chemistry and hematology parameter. To generate reference intervals, we used guidelines summarized in [11], which delineate statistical approaches based on sample size and distribution. To maintain a consistent statistical approach, we used nonparametric percentile methods to determine RIs for the whole sample, then RIs for each sex, age group, and rearing status, in which the upper and lower limits are defined by the 2.5th and 97.5th percentiles of the data for each parameter. Although the guidelines by Friedrichs et al. suggest the use of the robust method to calculate RIs in the current study for the infant and adult subgroups due to sample sizes, this yielded negative values for several of the parameters. Therefore, we opted to use the nonparametric method, which the guidelines state is acceptable [11]. Due to sample sizes below 40 in the geriatric category, the lower limit shown is the 2.5th percentile, while the upper limit is the upper range within the dataset. Analyses were performed in SPSS and R 4.5.0 [23] using the *referenceIntervals* package [24] to confirm findings.

**TABLE 2:** Descriptive statistics of serum chemistry and hematology parameters across all baboons in the sample.

Parameter	Outliers removed from the dataset	N	Range	Mean	SD	Median	95% Reference Interval
*Chem*							
ALB	2.7;2.7	528	2.9–5	4.26	0.33	4.30	3.4–4.7
Cl	None	530	95–114	107.04	2.51	107.00	102–112
CREA	None	530	0.22–1.35	0.61	0.20	0.57	0.3–1.1
K	None	530	2.8–4.9	3.50	0.32	3.50	3–4.2
Na	131;135;136	527	137–157	146.64	2.20	147.00	142–151
TP	None	530	5.1–8.3	6.73	0.48	6.70	5.8–7.7
ALT	192;190;166;142;118;110;99;97;96;96;91;90;89;86;85;84;83	513	7–80	34.43	12.44	32.00	16–66
ALP	None	530	60–2768	829.10	459	894.50	114–1730
AST	116;106;89	527	15–76	33.06	9.90	31.00	18–57
Ca	None	530	8.1–11.4	9.82	0.40	9.80	9–11
GLUC	257;170;166;163	526	40–154	85.83	18.25	83.50	57–132
GGT	None	530	16–111	54.66	16.86	53.00	26–90
LDH	1449;1389	528	128–985	371.10	155	336.00	173–786
TBIL	0.44;0.40	528	0.08–0.33	0.17	0.04	0.17	0.1–0.3
BUN	33;30;30	527	4–24	14.61	3.39	15.00	7.2–21
PHOS	None	530	1–8.9	4.82	1.50	4.95	1.8–7.5
CHOL	194	529	38–185	93.20	24.60	90.00	54–157
TRIG	158;148;138;138;134;132;129	523	18–122	51.72	17.83	49.00	24–98
CK	13 659;4942;3644;3628;2537;2406;2388;2302;1815;1738;1449;1410;1385;1325;1318;1277;170;1251;1219;1191;1064;990	508	43–946	316.63	157.69	274.50	126–736
FE	None	530	25–255	135.66	36.46	135.00	63–212
UIBC	None	530	85–437	243.61	48.29	244.00	145–341
AGAP	41;36;32;32;31;31;30;29;28	521	11–27	16.81	3.14	16.00	13–24
AGR		530	0.6–2.9	1.81	0.42	1.80	1.0–2.6
osmo	305;255;260	527	266–299	282.74	4.23	283.00	274–290
TIBC	572	529	290–535	378.90	38.50	377.00	309–469
GLOB	5.1	529	1.4–4.8	2.47	0.52	2.40	1.6–3.7
TCO2	11	529	12–35	26.00	3.54	27.00	17–31
*CBC*							
BASO	None	514	0.0–0.07	0.025	0.014	0.02	0.01–0.06
EOS	0.57;0.47;0.44;0.35;0.30;0.29;0.28;0.28;0.25;0.24 × 3	502	0.0–0.2	0.033	0.039	0.02	0–0.16
HCT	None	519	30–49	40	2.5	40	35–45
HGB	None	519	8.9–15	12	0.79	12	10.6–13.9
LYMP	None	514	3.1–80	29	18	25	6–69
LYMP%	None	510	0.4–6.5	2.2	1.1	1.9	0.8–5.1
MCH	8.67;8.26;6.90;6.78	519	22–29	26	1.2	26	23–28
MCHC	None	519	27–35	31	1.2	31	28–33
MCV	None	519	72–95	83	3.6	83	76–91
MONO	1.17;0.98;0.91;0.89;0.86	509	0.0–0.8	0.27	0.13	0.24	0.09–0.59
MPV	14.1	507	6.8–12	8.6	1.1	8.4	7.1–12
NEUT	23.9	513	1–18	6.4	3.9	5.4	1.3–16
NEUT%	None	519	0–95	66	20	71	24–92
PLT	None	508	90–698	362	80	351	235–547
RBC	None	519	3.5–5.7	4.8	0.31	4.8	4.2–5.5
RDW	None	519	11–16	13	0.83	13	12–15
WBC	26.5;25.16	517	2.7–23	9	3.7	8.2	3.9–18

*Note: N*, mean, standard deviation (SD), median, and reference interval (RI) calculated following removal of outliers show in the outlier column.

To examine differences across parameters as a function of viral status, we first matched each conventional baboon as closely as possible on age, sex, and rearing to SPF baboons, given the smaller sample size of conventional baboons. We then used a multivariate analysis of variance (MANOVA) with parameters as the dependent variables and SPF status (SPF vs. conventional) as the independent variable. Although there were statistically significant differences across parameters between SPF and conventional baboons (see results below), we opted to include conventional baboons in the remaining analyses for a few reasons: (1) the differences were small in magnitude; (2) conventional baboons are some of the oldest baboons in the colony and therefore provide important data for the geriatric age category; and (3) excluding conventional baboons would limit the utility of the RIs for any baboons that are not SPF. Although we did not calculate separate reference intervals for SPF and conventional baboons due to small sample sizes (less than 30), we do report means, standard errors, and the statistical results below.

To examine differences in parameters as a function of sex, rearing, and age category, we used MANOVAs with sex, age category, and rearing status as the between‐subjects factors and each parameter within the chemistry and hematology datasets as the dependent variables. We then calculated the 95% reference intervals as the 2.5th percentile as the lower limit and 97.5th percentile as the upper limit of each parameter across sex, age, and rearing categories. Parameters showing significant differences from the MANOVAs are plotted as mean ± SEM against the overall RI. That is, the *y*‐axis range includes, as closely as possible, the 95% RI for the entire sample for that parameter. We chose this method of visual presentation to illustrate the statistical differences across groups within their reference interval.

We generated growth curves by plotting weight against age using a generalized additive model and plotting the standard error using ggplot in R (v. 4.5.1) [23, 25]. Curves were created to depict growth across all ages showing differences between males and females, early differences (0–36 months) in weight development between mother‐ and nursery‐reared baboons, and differences in growth as a function of rearing across the full age range separately for males and females.

## 3 Results

Across the full dataset, including all animals across viral status, age, rearing, and sex, we report the range, mean, SEM, median, and the overall RI for each serum chemistry and hematology parameter in Table 2.

**FIGURE 1:**
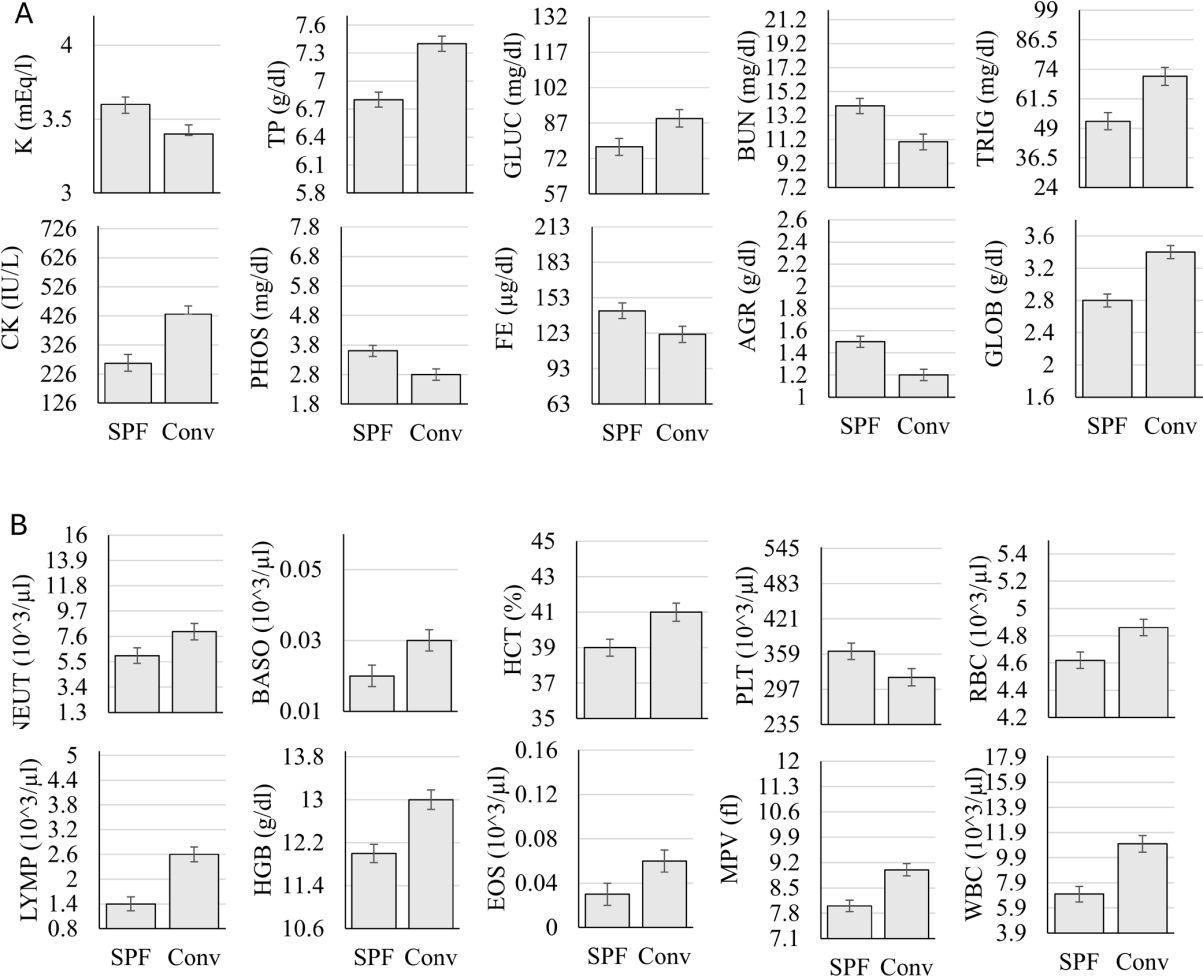
Statistically significant differences (*p* < 0.05) in (A) serum chemistry parameters and (B) hematology parameters. The *y*‐axis in each figure represents the overall reference interval for that parameter.

The MANOVA examining the effects of viral status across parameters using age‐, sex‐, and rearing‐matched subjects showed that 10 of the 17 (58.8%) hematology parameters and 10 of the 27 (37%) chemistry parameters were significantly different across SPF and conventional baboons (*p* < 0.05; Table 3). Significant differences are plotted in Figure 1.

**TABLE 3:** MANOVA results: Differences in parameters as a function of SPF status.

Parameter	SPF mean ± SE	Conventional mean ± SE	*F* (1,48)	*p*
*Chem*				
ALB	4 ± 0.08	4 ± 0.08	0	0.89
Cl	107 ± 0.44	108 ± 0.45	1	0.26
CREA	0.79 ± 0.04	0.8 ± 0.04	0	0.85
K	3.6 ± 0.05	3.4 ± 0.06	7	0.01
Na	147 ± 0.34	147 ± 0.35	0	0.84
TP	6.8 ± 0.08	7.4 ± 0.08	26	0.00
ALT	35 ± 3.2	33 ± 3.3	0	0.69
ALP	224 ± 31	233 ± 32.3	0	0.83
AST	26 ± 1	28 ± 1.1	2	0.15
Ca	9.5 ± 0.07	9.6 ± 0.07	2	0.18
GLUC	77 ± 3.6	89 ± 3.7	5	0.03
GGT	36 ± 1.8	35 ± 1.9	0	0.95
LDH	311 ± 24	344 ± 25	1	0.34
TBIL	0.17 ± 0.01	0.17 ± 0.01	0	0.81
BUN	14 ± 0.64	11 ± 0.66	11	0.00
PHOS	3.6 ± 0.19	2.8 ± 0.19	9	0.00
CHOL	82 ± 3.5	90 ± 3.7	3	0.10
TRIG	52 ± 3.6	71 ± 3.8	13	0.00
CK	263 ± 30	432 ± 28	19	0.00
FE	142 ± 6.6	122 ± 6.9	4	0.04
UIBC	255 ± 9.5	262 ± 9.8	0	0.61
AGAP	16 ± 0.49	16 ± 0.51	0	0.98
AGR	1.5 ± 0.05	1.2 ± 0.05	14	0.00
osmo	282 ± 0.68	282 ± 0.71	0	0.82
TIBC	397 ± 8	384 ± 8.3	1	0.27
GLOB	2.8 ± 0.08	3.4 ± 0.08	25	0.00
TCO2	28 ± 0.53	27 ± 0.55	1	0.24

Parameter	SPF mean ± SE	Conventional mean ± SE	*F* (1,57)	*p*
*CBC*				
BASO	0.02 ± 0.003	0.03 ± 0.003	17	0.00
EOS	0.03 ± 0.01	0.06 ± 0.01	6	0.02
HCT	39 ± 0.48	41 ± 0.51	9	0.00
HGB	12 ± 0.17	13 ± 0.18	5	0.03
LYMP%	22 ± 2.3	26 ± 2.4	1	0.26
LYMP	1.4 ± 0.17	2.6 ± 0.18	23	0.00
MCH	26 ± 0.24	26 ± 0.25	0	0.88
MCHC	31 ± 0.24	31 ± 0.25	0	0.59
MCV	84 ± 0.58	84 ± 0.61	0	0.74
MONO	0.28 ± 0.03	0.3 ± 0.03	0	0.66
MPV	8 ± 0.16	9 ± 0.17	29	0.00
NEUT	6 ± 0.65	8 ± 0.68	7	0.01
NEUT%	73 ± 2.4	70 ± 2.6	1	0.45
PLT	364 ± 14.4	318 ± 15.2	5	0.03
RBC	4.62 ± 0.06	4.86 ± 0.06	8	0.01
RDW	13 ± 0.13	13 ± 0.14	0	0.99
WBC	7 ± 0.62	11 ± 0.66	16	0.00

Abbreviations: SE, standard error of the mean; SPF, specific pathogen free.

**FIGURE 2:**
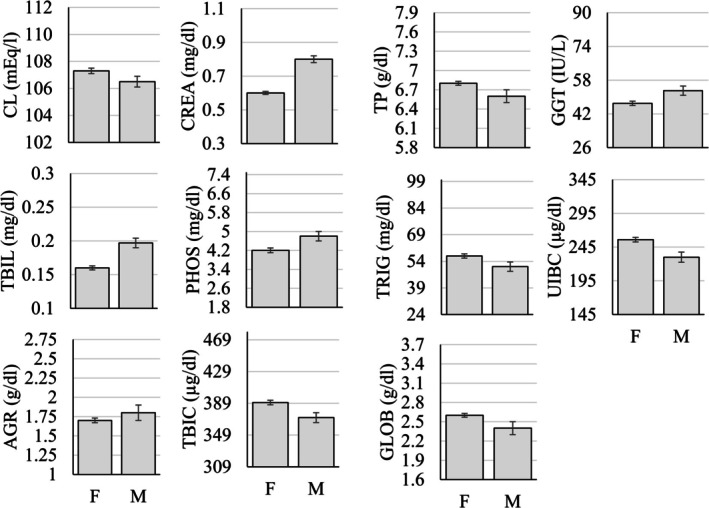
Statistically significant differences (*p* < 0.05) in serum chemistry values as a function of sex (F, Female; M, Male). The *y*‐axis in each figure represents the overall reference interval for that parameter.

**FIGURE 3:**
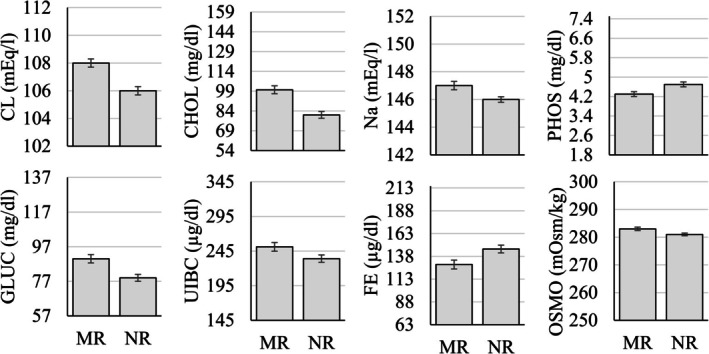
Statistically significant differences (*p* < 0.05) in serum chemistry values as a function of rearing (MR, Mother‐reared; NR, Nursery‐reared). The *y*‐axis in each figure represents the overall reference interval for that parameter.

**FIGURE 4:**
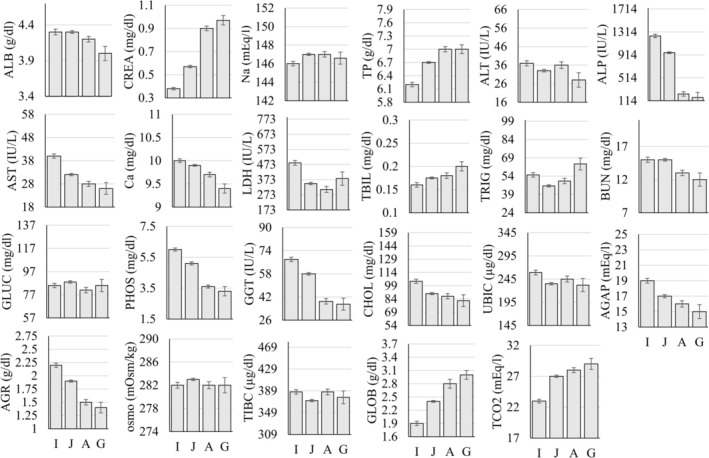
Statistically significant differences (*p* < 0.05) in serum chemistry values as a function of the age group (A, Adult; G, Geriatric; I, Infant; J, Juvenile). The *y*‐axis in each figure represents the overall reference interval for that parameter.

The MANOVA examining differences in serum chemistry parameters as a function of sex, age group (infant, juvenile, adult, geriatric), and rearing status (mother‐ vs. nursery‐reared) showed that 11 of the 27 (~41%) parameters were significantly different as a function of sex (Figure 2), 8 of the 27 (~30%) were significantly different as a function of rearing (Figure 3), and all but three of the 27 (~89%) were significantly different as a function of the age group (Figure 4 and Table 4).

**TABLE 4:** MANOVA results: Differences in serum chemistry parameters as a function of sex, age group, and rearing.

Parameter	Sex				Age category						Rearing			
	Female (*n* = 259) mean ± SE	Male (*n* = 205) mean ± SE	*F* (1, 448)	*p*	Infant (0–12 mos; *n* = 78) mean ± SE	Juvenile (1–5 years; *n* = 280) mean ± SE	Adult (5–15 years; *n* = 80) mean ± SE	Geriatric (> 15 years; *n* = 26) mean ± SE	*F* (1, 448)	*p*	MR (*n* = 232) mean ± SE	NR (*n* = 232) mean ± SE	*F* (1, 448)	*p*
ALB	4.2 ± 0.02	4.2 ± 0.1	0	0.50	4.3 ± 0.04	4.3 ± 0.02	4.2 ± 0.04	4.0 ± 0.1	7	0.00	4.214 ± 0.04	4.178 ± 0.04	0	0.52
Cl	107.3 ± 0.2	106.5 ± 0.4	4	0.04	107 ± 0.3	107 ± 0.1	107 ± 0.3	106 ± 0.7	1	0.24	108 ± 0.3	106 ± 0.3	11	0.00
CREA	0.6 ± 0.009	0.8 ± 0.02	36	0.00	0.38 ± 0.01	0.57 ± 0.01	0.90 ± 0.02	0.97 ± 0.04	247	0.00	0.7 ± 0.02	0.7 ± 0.01	0	0.89
K	3.5 ± 0.02	3.5 ± 0.05	0	0.59	3.5 ± 0.03	3.5 ± 0.02	3.6 ± 0.04	3.4 ± 0.1	2	0.09	3.4 ± 0.04	3.5 ± 0.04	2	0.19
Na	147 ± 0.2	146 ± 0.3	1	0.45	146 ± 0.23	147 ± 0.12	147 ± 0.3	146.6 ± 0.65	3	0.02	147 ± 0.3	146 ± 0.2	11	0.00
TP	6.8 ± 0.03	6.6 ± 0.1	5	0.02	6.2 ± 0.05	6.7 ± 0.02	7.0 ± 0.06	7.0 ± 0.1	49	0.00	6.8 ± 0.1	6.7 ± 0.05	2	0.20
ALT	35 ± 1.0	32 ± 2.1	2	0.15	37 ± 1.4	33 ± 0.7	36 ± 1.8	28 ± 3.9	3	0.02	32 ± 1.8	35 ± 1.5	2	0.17
ALP	622 ± 21.4	677 ± 46.5	1	0.28	1243 ± 31.6	951 ± 16.2	233 ± 39.6	171 ± 87.4	160	0.00	690 ± 39.0	608 ± 33.0	3	0.11
AST	31 ± 0.6	32 ± 1.3	0	0.95	40 ± 0.9	32 ± 0.5	28 ± 1.1	26 ± 2.5	33	0.00	31 ± 1.1	32 ± 0.9	1	0.48
Ca	9.8 ± 0.03	9.7 ± 0.1	0	0.57	10.0 ± 0.04	9.9 ± 0.02	9.7 ± 0.05	9.4 ± 0.1	11	0.00	9.8 ± 0.1	9.7 ± 0.04	2	0.12
GLUC	83 ± 1.3	86 ± 2.8	0	0.50	85 ± 1.9	88 ± 1.0	81 ± 2.4	85 ± 5.3	3	0.05	90 ± 2.4	79 ± 2.0	14	0.00
GGT	47 ± 1.0	53 ± 2.2	6	0.01	68 ± 1.5	58 ± 0.8	39 ± 1.9	37 ± 4.1	56	0.00	50 ± 1.8	51 ± 1.6	0	0.75
LDH	376 ± 10.7	383 ± 23.4	0	0.78	484 ± 15.9	347 ± 8.1	308 ± 19.9	380 ± 43.9	23	0.00	388 ± 19.6	371 ± 16.6	0	0.49
TBIL	0.16 ± 0.003	0.197 ± 0.007	20	0.00	0.16 ± 0.005	0.175 ± 0.002	0.18 ± 0.006	0.2 ± 0.01	5	0.00	0.2 ± 0.01	0.2 ± 0.01	0	0.77
BUN	14 ± 0.2	13 ± 0.5	2	0.12	15 ± 0.4	15 ± 0.2	13 ± 0.4	12 ± 1.0	12	0.00	13 ± 0.4	14 ± 0.4	4	0.06
PHOS	4.2 ± 0.1	4.8 ± 0.2	12	0.00	6.0 ± 0.1	5.1 ± 0.1	3.6 ± 0.1	3.3 ± 0.3	65	0.00	4.3 ± 0.1	4.7 ± 0.1	5	0.02
CHOL	90 ± 1.6	91 ± 3.5	0	0.98	104 ± 2.4	90 ± 1.2	87 ± 3.0	82 ± 6.6	12	0.00	100 ± 3.0	81 ± 2.5	25	0.00
TRIG	57 ± 1.2	51 ± 2.6	5	0.03	55 ± 1.8	46 ± 0.9	50 ± 2.2	64 ± 4.9	11	0.00	55 ± 2.2	53 ± 1.8	0	0.57
CK	337 ± 12.3	284 ± 26.7	3	0.07	334 ± 18.2	307 ± 9.3	297 ± 22.7	304 ± 50.2	1	0.54	300 ± 22.5	321 ± 19.0	1	0.46
FE	134 ± 2.7	141 ± 5.8	1	0.29	128 ± 4.0	136 ± 2.0	142 ± 5.0	145 ± 11.0	2	0.10	129 ± 4.9	146 ± 4.2	7	0.01
UIBC	256 ± 3.4	230 ± 7.5	10	0.00	259 ± 5.1	235 ± 2.6	245 ± 6.4	232 ± 14.0	7	0.00	251 ± 6.3	234 ± 5.3	4	0.04
AGAP	17 ± 0.2	17 ± 0.5	0	0.83	19 ± 0.3	17 ± 0.2	16 ± 0.4	15 ± 0.9	25	0.00	17 ± 0.4	17 ± 0.3	0	1.00
AGR	1.7 ± 0.03	1.8 ± 0.1	5	0.03	2.2 ± 0.04	1.9 ± 0.02	1.5 ± 0.05	1.4 ± 0.1	63	0.00	1.7 ± 0.05	1.8 ± 0.04	1	0.29
osmo	283 ± 0.3	282 ± 0.7	1	0.26	282 ± 0.5	283 ± 0.2	282 ± 0.6	282 ± 1.3	3	0.02	283 ± 0.6	281 ± 0.5	12	0.00
TIBC	390 ± 2.9	371 ± 6.2	8	0.01	387 ± 4.2	371 ± 2.2	387 ± 5.3	377 ± 11.7	6	0.00	380 ± 5.2	380 ± 4.4	0	1.00
GLOB	2.6 ± 0.03	2.4 ± 0.1	8	0.01	1.9 ± 0.05	2.4 ± 0.02	2.8 ± 0.1	3.0 ± 0.1	60	0.00	2.6 ± 0.1	2.5 ± 0.05	1	0.42
TCO2	26 ± 0.2	27 ± 0.5	1	0.42	23 ± 0.3	27 ± 0.2	28 ± 0.4	29 ± 0.9	52	0.00	26 ± 0.4	26 ± 0.3	0	0.77

**FIGURE 5:**
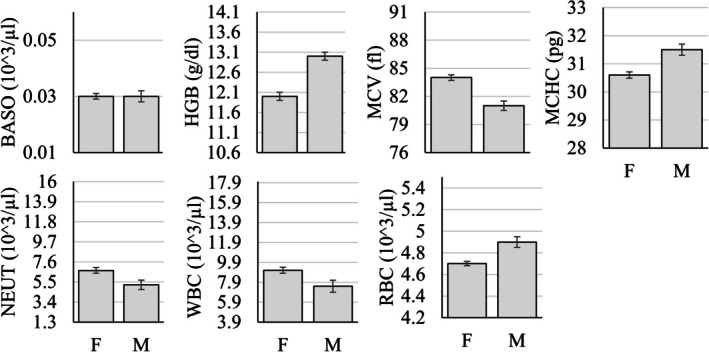
Statistically significant differences (*p* < 0.05) in hematology values as a function of sex (F, Female; M, Male). The *y*‐axis in each figure represents the overall reference interval for that parameter.

**FIGURE 6:**
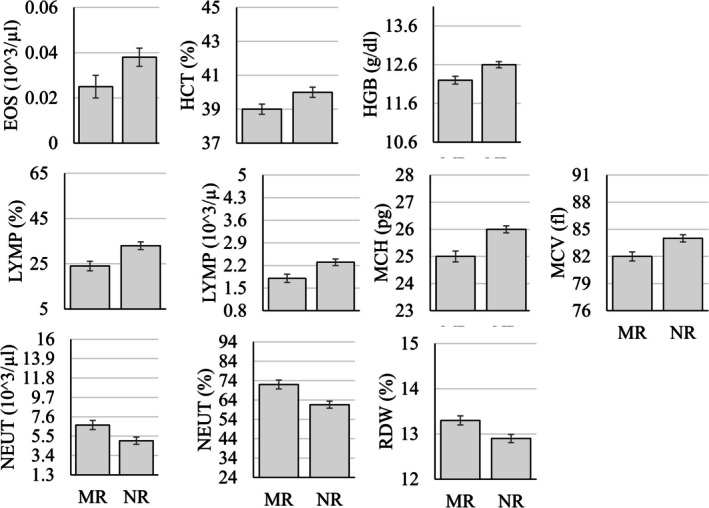
Statistically significant differences (*p* < 0.05) in hematology values as a function of rearing (MR, Mother‐reared; NR, Nursery‐reared). The *y*‐axis in each figure represents the overall reference interval for that parameter.

**FIGURE 7:**
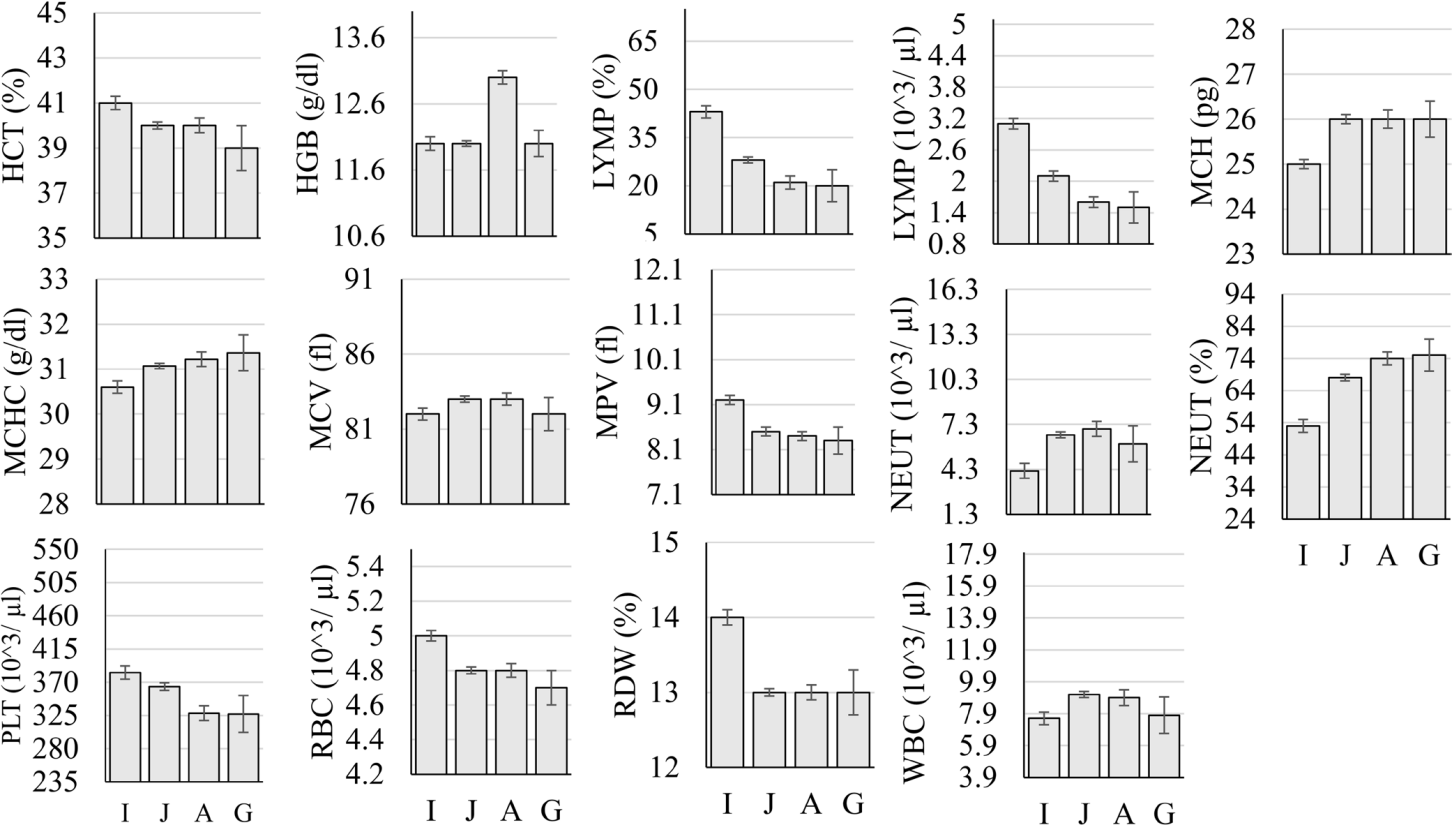
Statistically significant differences (*p* < 0.05) in hematology values as a function of the age group (A, Adult; G, Geriatric; I, Infant; J, Juvenile). The y‐axis in each figure represents the overall reference interval for that parameter.

The MANOVA examining differences in hematology parameters as a function of sex, age group (infant, juvenile, adult, geriatric), and rearing status (mother‐ vs. nursery‐reared) showed that 7 of the 17 (~41%) parameters were significantly different as a function of sex (Figure 5), 10 of the 17 (~59%) were significantly different as a function of rearing (Figure 6), and all but three of the 17 (~82%) were significantly different as a function of the age group (Figure 7 and Table 5).

**TABLE 5:** MANOVA results: Differences in hematological parameters as a function of sex, age group, and rearing.

Parameter	Sex				Age category						Rearing			
	Female (*n* = 272)	Male (*n* = 212)	*F* (1, 468)	*p*	Infant (0–12mos; *n* = 79)	Juvenile (1–5 years; *n* = 282)	Adult (5–15 years; *n* = 93)	Geriatric (> 15 years; *n* = 30)	*F* (1, 468)	*p*	MR (*n* = 259)	NR (*n* = 225)	*F* (1, 468)	*p*
BASO	0.03 ± 0.001	0.03 ± 0.002	7	0.01	0.026 ± 0.002	0.024 ± 0.001	0.023 ± 0.002	0.018 ± 0.005	1	0.36	0.023 ± 0.002	0.023 ± 0.002	0	0.97
EOS	0.03 ± 0.003	0.03 ± 0.006	1	0.38	0.03 ± 0.004	0.035 ± 0.002	0.031 ± 0.005	0.028 ± 0.011	1	0.65	0.025 ± 0.005	0.038 ± 0.004	4	0.04
HCT	40 ± 0.23	40 ± 0.4	1	0.35	41 ± 0.3	40 ± 0.15	40 ± 0.33	39 ± 1	4	0.00	39 ± 0.3	40 ± 0.3	6	0.02
HGB	12 ± 0.1	13 ± 0.1	12	0.00	12 ± 0.1	12 ± 0.04	13 ± 0.1	12 ± 0.2	3	0.04	12.2 ± 0.1	12.6 ± 0.08	9	0.00
LYMP%	27 ± 1.5	29 ± 2.5	1	0.42	43 ± 1.9	28 ± 0.9	21 ± 2	20 ± 5	25	0.00	24 ± 2.1	33 ± 1.7	12	0.00
LYMP	2.1 ± 0.1	2.0 ± 0.2	0	0.60	3.1 ± 0.1	2.1 ± 0.1	1.6 ± 0.1	1.5 ± 0.3	28	0.00	1.8 ± 0.13	2.3 ± 0.1	8	0.00
MCH	26 ± 0.1	26 ± 0.2	0	0.58	25 ± 0.1	26 ± 0.1	26 ± 0.2	26 ± 0.4	10	0.00	25 ± 0.2	26 ± 0.13	13	0.00
MCHC	30.6 ± 0.11	31.5 ± 0.2	16	0.00	30.6 ± 0.14	31.1 ± 0.06	31.22 ± 0.16	31.36 ± 0.4	3	0.02	31 ± 0.16	31 ± 0.13	1	0.46
MCV	84 ± 0.3	81 ± 0.5	18	0.00	82 ± 0.4	83 ± 0.2	83 ± 0.4	82 ± 1.1	3	0.02	82 ± 0.5	84 ± 0.4	11	0.00
MONO	0.3 ± 0.0	0.2 ± 0.0	2	0.14	0.2 ± 0.02	0.3 ± 0.01	0.3 ± 0.02	0.3 ± 0.04	1	0.27	0.24 ± 0.02	0.28 ± 0.01	2	0.13
MPV	8.7 ± 0.1	8.5 ± 0.2	2	0.20	9.2 ± 0.1	8.5 ± 0.1	8.4 ± 0.1	8.3 ± 0.3	8	0.00	8.6 ± 0.15	8.6 ± 0.12	0	0.84
NEUT	6.7 ± 0.4	5.2 ± 0.6	5	0.03	4.2 ± 0.5	6.6 ± 0.2	7.0 ± 0.5	6.0 ± 1.2	8	0.00	6.7 ± 0.5	5 ± 0.4	7	0.01
NEUT%	69 ± 1.6	66 ± 2.6	1	0.42	53 ± 2	68 ± 1	74 ± 2	75 ± 5	23	0.00	72 ± 2.3	61.6 ± 1.8	14	0.00
PLT	360 ± 7	341 ± 12	2	0.18	383 ± 9	364 ± 5	328 ± 10	327 ± 25	6	0.00	361 ± 11	340 ± 9	2	0.13
RBC	4.7 ± 0.02	4.9 ± 0.05	14	0.00	5.0 ± 0.03	4.8 ± 0.02	4.8 ± 0.04	4.7 ± 0.1	10	0.00	4.8 ± 0.04	4.8 ± 0.03	0	0.83
RDW	13 ± 0.1	13 ± 0.1	0	0.75	14 ± 0.1	13 ± 0.05	13 ± 0.1	13 ± 0.3	7	0.00	13.3 ± 0.1	12.9 ± 0.09	6	0.02
WBC	9.1 ± 0.3	7.5 ± 0.6	6	0.02	7.6 ± 0.4	9.1 ± 0.2	8.9 ± 0.5	7.8 ± 1.14	3	0.02	8.8 ± 0.49	7.6 ± 0.39	3	0.06

**FIGURE 8:**
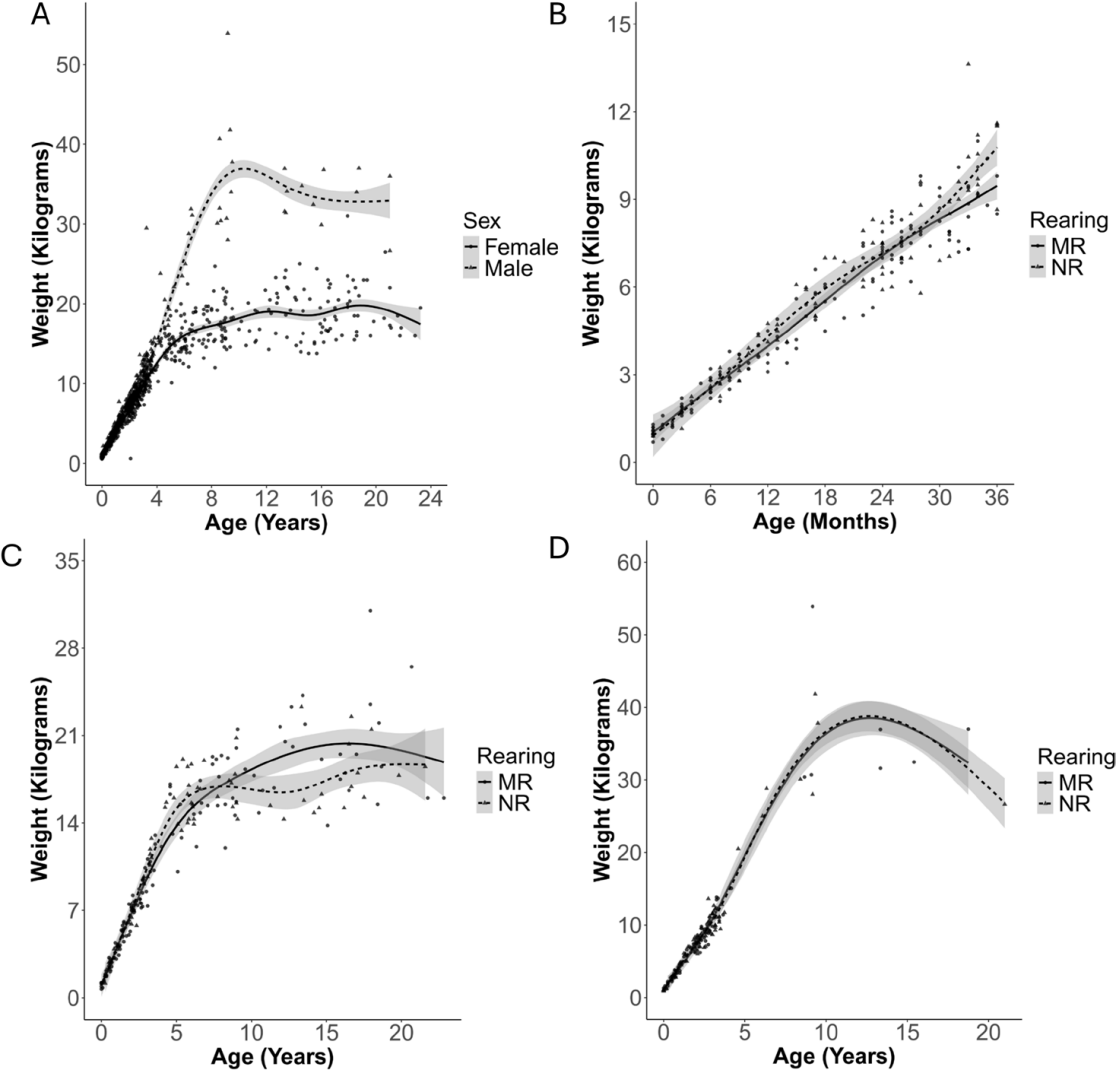
Growth curves for (A) males and females across all ages 0–23 years and (B) mother‐reared (MR) and nursery‐reared (NR) baboons among juveniles (0–36 months), including males and females; (C) MR and NR females only across all ages; and (D) MR and NR males only across all ages.

Mean weight ± standard error is plotted according to age and sex (Figure 8A). The growth curve showing mother‐ vs. nursery‐reared baboons 0–36 months of age is shown in Figure 8B. Mean weight ± standard error is plotted according to age and rearing history, separately for females (Figure 8C) and males (Figure 8D).

## 4 Discussion

In the current study, we aimed to create a comprehensive and robust source for captive olive baboon hematological and blood chemistry values, along with reference intervals (RIs) and growth curves. Utilizing moderate to large sample sizes, we present (1) multiple measures of central tendency and variation across parameters, (2) statistical results regarding differences as a function of important demographic variables, including viral status, age group, sex, and rearing, and (3) RIs across the entire sample as well as within multiple subgroups. Additionally, we provide growth curves using the largest dataset to date, across age, sex, and rearing categories for captive olive baboons. These data will serve the biomedical and veterinary care communities that house olive baboons, a common lab animal species.

To our knowledge, this is the first study to use full, standard hematology and serum chemistry panels across a wide age range to directly compare SPF‐18 and conventional baboon parameters. Using a matched‐subject design, we found that several parameters were different as a function of viral status. While SPF‐18 baboons showed significantly higher levels of potassium, blood urea nitrogen, phosphate, iron, albumin to globulin ratio, and platelets, they showed lower levels of total protein glucose, triglycerides, creatinine kinase, globulin, neutrophils, basophils, hematocrit, red blood cell counts, lymphocyte counts, hemoglobin, eosinophils, mean corpuscular volume, and white blood cell counts compared to their conventional counterparts. Some of these results may be unsurprising given that conventional baboons have a higher viral burden and, therefore, are likely to have higher immunological activation, leading to elevated parameters such as white blood cells. When applying these reference intervals to non‐SPF baboons, it may be useful to consider them alongside statistical findings related to viral status. As an example, given that lymphocyte counts were statistically higher in conventional animals, and the majority of our dataset consisted of SPF baboons, it is reasonable to infer that lymphocyte reference values for conventional or wild baboons may be higher than those reported here.

Hematology and serum chemistry values also differed as a function of sex and age group. The finding that parameters differed according to sex is unsurprising, given biological differences between the sexes and previous literature demonstrating such differences across primate taxa [26, 27]. Differences as a function of age are also unsurprising, given the development of physiological systems from infancy to old age. For example, geriatric baboons are more likely to have lower hematocrit and red blood cell counts, indicative of anemia, which is a common age‐related change also observed in humans [28]. Furthermore, our results confirm that ranges for infants and juveniles are wider than for adults, indicating that separate reference ranges should be used for each age group [12].

We also found differences in parameters as a function of rearing. While mother‐reared baboons showed higher mean values for neutrophils (absolute counts and percent), red cell distribution width, chloride, cholesterol, sodium, glucose, UBIC, and osmolality, nursery‐reared baboons showed higher mean values for eosinophils, hematocrit, hemoglobin, lymphocytes (absolute counts and percents), mean corpuscular hemoglobin, mean corpuscular volume, phosphate, and iron. Nursery rearing has been shown to affect a variety of health systems across primate species. Of most importance to hematological and chemistry values are the effects of stress‐related [29, 30, 31, 32] and immune [33, 34, 35] systems. For example, nursery‐reared rhesus macaques show altered HPA‐axis activity and responsiveness [29, 36], which may lead to dysregulated stress‐induced lymphocyte responses as well as other downstream changes in hematology and chemistry [37]. Additionally, nursery‐reared primates tend to have higher lymphocyte counts as well as lower neutrophil to lymphocyte ratios (NLRs), compared to their mother‐reared counterparts [20, 38, 39]. These effects of nursery rearing may be due to altered maturation of lymphocyte subsets resulting from early deprivation of maternal microbial exposure and social contact during critical periods for immune development [33, 38, 40]. The differences in parameters reflecting immunological, metabolic, renal, and hematologic function found in the current study may further support the effects of the early rearing environment on physiological and homeostatic systems during critical developmental periods. However, it's important to emphasize that the levels of these parameters in nursery‐reared individuals are within normal limits and reference ranges reported previously and herein. As such, although differences between mother‐reared and nursery‐reared individuals may be statistically significant, they may not be clinically relevant (see below).

In the current study, we observed numerous statistically significant differences in hematology and serum chemistry parameters across viral status, sex, rearing, and age groups. An important distinction must be made between statistical significance and clinical significance. While statistical significance indicates a low probability that an observed difference is due to chance, clinical or biological significance refers to whether the magnitude of the difference is meaningful in a physiological or health‐related context. In the case of the current findings, while some statistically significant differences may reflect meaningful biological variation, we would argue that others may be of little clinical relevance. For example, the significant difference in RBC between SPF‐18 and conventional baboons is just 0.24 and likely reached statistical significance due to the low standard error within groups. A difference of this magnitude is unlikely to reflect a pathological state or to impact clinical interpretation in a meaningful way. As such, we recommend interpreting findings in the contexts of both clinical and statistical significance. This is particularly important for large datasets in which small differences across groups can achieve statistical significance due to high statistical power.

The growth curves presented in this study show steady growth in females until about 6 years of age and in males until about 8 years of age. Upon maturity (around age 6), males, on average, weighed approximately twice as much as females. These results are consistent with previous baboon growth and weight data [17, 18, 19]. However, both males and females in our sample seem to weigh more, on average, than captive and wild baboons reported previously. For example, male baboons were reported to weigh an average of approximately 21–28 kg and females an average of approximately 12–15 kg [9, 17, 18, 19], whereas our data show an average of 35 kg and 17 kg, respectively. This may be due, in part, to the incidence of obesity in our sample. Clinical data (not included here) showed that, of the 107 adult baboons sampled for BCS during the fall 2023 physical exams, approximately 14% were classified as having a BCS of 4 or greater (indicating overweight or obese). Furthermore, adult baboons classified as overweight or obese weighed 5.74 kg (females) and 11.6 kg (males) more than their normal BCS counterparts (data not included here). However, of note, several baboons, both males and females, that were similar in weight to those classified as overweight or obese actually had normal BCSs (BCS of 3). These baboons were noted to be large in stature in their exam records; unfortunately, we do not currently have anthropometric data such as body length to qualitatively substantiate these qualitative notes in the record. Overall, the higher weights compared to previously published literature may be due to a combination of the incidence of overweight and obese individuals artificially increasing the mean, as well as larger statured females in general. Future research should combine weight, body condition scores, and crown to rump measurements for qualitative assessments of growth according to body mass, rather than simply weight, in captive baboons.

Baboons are becoming more widely used as models for aging and aging‐related diseases and disorders (including neuropathology related to Alzheimer's disease and related dementias as well as cardiac and kidney xenotransplantation) [1, 6, 7]. As indicated by the relatively larger standard errors of parameters within the geriatric group, a larger sample size of geriatric baboons is needed to better define RIs in this age group. Furthermore, we did not examine differences in subgroups (e.g., male adults vs. female adults vs. male juveniles vs. female juveniles) due to the smaller sample sizes this would require. As such, future research should examine differences in parameters across these nested subgroups. Lastly, environmental factors that can affect hematology and serum chemistry parameters that were not examined in the current study warrant further investigation, including stress caused by restraint prior to sedation [20, 41], housing type (laboratory: corrals, Primadomes, run‐housing, indoor housing, etc.; zoo‐settings; wild populations), group size, diet, and variations in physical conditions (e.g., obesity) [41], to name a few.

## Conflicts of Interest

The authors declare no conflicts of interest.

## Data Availability

The data that support the findings of this study are available on request from the corresponding author. The data are not publicly available due to privacy or ethical restrictions.
